# Development of constrictional microchannels and the recurrent neural network in single-cell protein analysis

**DOI:** 10.3389/fbioe.2023.1195940

**Published:** 2023-05-03

**Authors:** Ting Zhang, Xiao Chen, Deyong Chen, Junbo Wang, Jian Chen

**Affiliations:** ^1^ State Key Laboratory of Transducer Technology, Aerospace Information Research Institute, Chinese Academy of Sciences, Beijing, China; ^2^ School of Future Technology, University of Chinese Academy of Sciences, Beijing, China; ^3^ School of Electronic, Electrical and Communication Engineering, University of Chinese Academy of Sciences, Beijing, China

**Keywords:** biosensors, single-cell proteomic analysis, quantitative flow cytometry, constrictional microchannel, recurrent neural network

## Abstract

**Introduction:** As the golden approach of single-cell analysis, fluorescent flow cytometry can estimate single-cell proteins with high throughputs, which, however, cannot translate fluorescent intensities into protein numbers.

**Methods:** This study reported a fluorescent flow cytometry based on constrictional microchannels for quantitative measurements of single-cell fluorescent levels and the recurrent neural network for data analysis of fluorescent profiles for high-accuracy cell-type classification.

**Results:** As a demonstration, fluorescent profiles (e.g., FITC labeled β-actin antibody, PE labeled EpCAM antibody and PerCP labeled β-tubulin antibody) of individual A549 and CAL 27 cells were firstly measured and translated into protein numbers of 0.56 ± 0.43 × 10^4^, 1.78 ± 1.0^6^ × 10^6^ and 8.11 ± 4.89 × 10^4^ of A549 cells (n_cell_ = 10232), and 3.47 ± 2.45 × 10^4^, 2.65 ± 1.19 × 10^6^ and 8.61 ± 5.25 × 10^4^ of CAL 27 cells (n_cell_ = 16376) based on the equivalent model of the constrictional microchannel. Then, the feedforward neural network was used to process these single-cell protein expressions, producing a classification accuracy of 92.0% for A549 vs. CAL 27 cells. In order to further increase the classification accuracies, as a key subtype of the recurrent neural network, the long short-term memory (LSTM) neural network was adopted to process fluorescent pulses sampled in constrictional microchannels directly, producing a classification accuracy of 95.5% for A549 vs. CAL 27 cells after optimization.

**Discussion:** This fluorescent flow cytometry based on constrictional microchannels and recurrent neural network can function as an enabling tool of single-cell analysis and contribute to the development of quantitative cell biology.

## 1 Introduction

Single-cell protein analysis can identify and analyze rare but key single cells from large populations, thereby facilitating the studies of cell heterogeneities (Heath et al., 2016; Nissen et al., 2022), which can contribute to early diagnosis, precision treatment, and drug development of diseases (Labib and Kelley, 2021).

As the golden approach, flow cytometry estimates single-cell protein expressions by measuring fluorescent levels of travelling single cells bound with antibodies with fluorescent probes (Huang et al., 2020; Tian et al., 2020; Yin et al., 2020). Leveraging calibration beads with predefined numbers of membrane proteins, specific numbers of membrane proteins are obtained based on fluorescent flow cytometry, which, however, cannot quantify cytoplasmic proteins because of lacking corresponding calibration beads with well-regulated internal protein numbers (Wang et al., 2016; Mizrahi et al., 2018). Lately, mass cytometry has realized high multiplexed detection of proteins, where single cells are bound with distinct transition element isotope-labeled antibodies, which, however, can only report relative intensities rather than specific numbers of targeted proteins because of lacking effective calibration methods (Mavropoulos et al., 2017; Ajami et al., 2018; Han et al., 2018; Ali et al., 2020).

Because of the dimensional comparison with biological cells, microfluidics has become a key technology to analyze single-cell proteins (Chen et al., 2019). More specifically, microfluidic large arrays were developed to quantify both intracellular and membrane proteins of single cells (Papp et al., 2017; Li et al., 2018a; Armbrecht et al., 2019). In these microfluidic platforms, single cells were distributed individually in microwells and the proteins under measurements were captured by antibodies coated beneath and estimated based on fluorescent intensities. However, different from flow cytometry, these microfluidic arrays cannot process single cells within fluid flow, leading to compromised throughputs.

Lately, a microfluidic flow cytometer based on a constrict (a microchannel with a cross-sectional area smaller than biological cells) was reported to estimate targeted proteins of single cells, where the constrict structure functioned as a calibration model of transferring preliminary fluorescent intensities into the protein numbers (Li et al., 2017; Liu et al., 2020). However, this approach was not applicable to quantification of proteins with uneven distributions within cells and may cause channel blockage with compromised throughputs.

Aimed to deal with the aforementioned problems, in this paper, the cross-sectional area of the constrict was enlarged to be marginally larger than the biological cells, enabling the collection of fluorescent pulses of stained single cells traveling through constrictional microchannels without blockage. Then, based on the volume equivalence between cells and calibration solutions following through constrictional microchannels, preliminary fluorescent pulses were translated into protein expressions at the single-cell level, which were further used for cell-type classification based on the feedforward neural network. In a second approach, without quantifying single-cell protein levels, preliminary fluorescent pulses measured by constrictional microchannels were processed by the long short-term memory (LSTM) neural network, a key subtype of the recurrent neural network, producing accuracies of cell-type classification for comparison.

## 2 Materials and methods

### 2.1 Working mechanism

**FIGURE 1:**
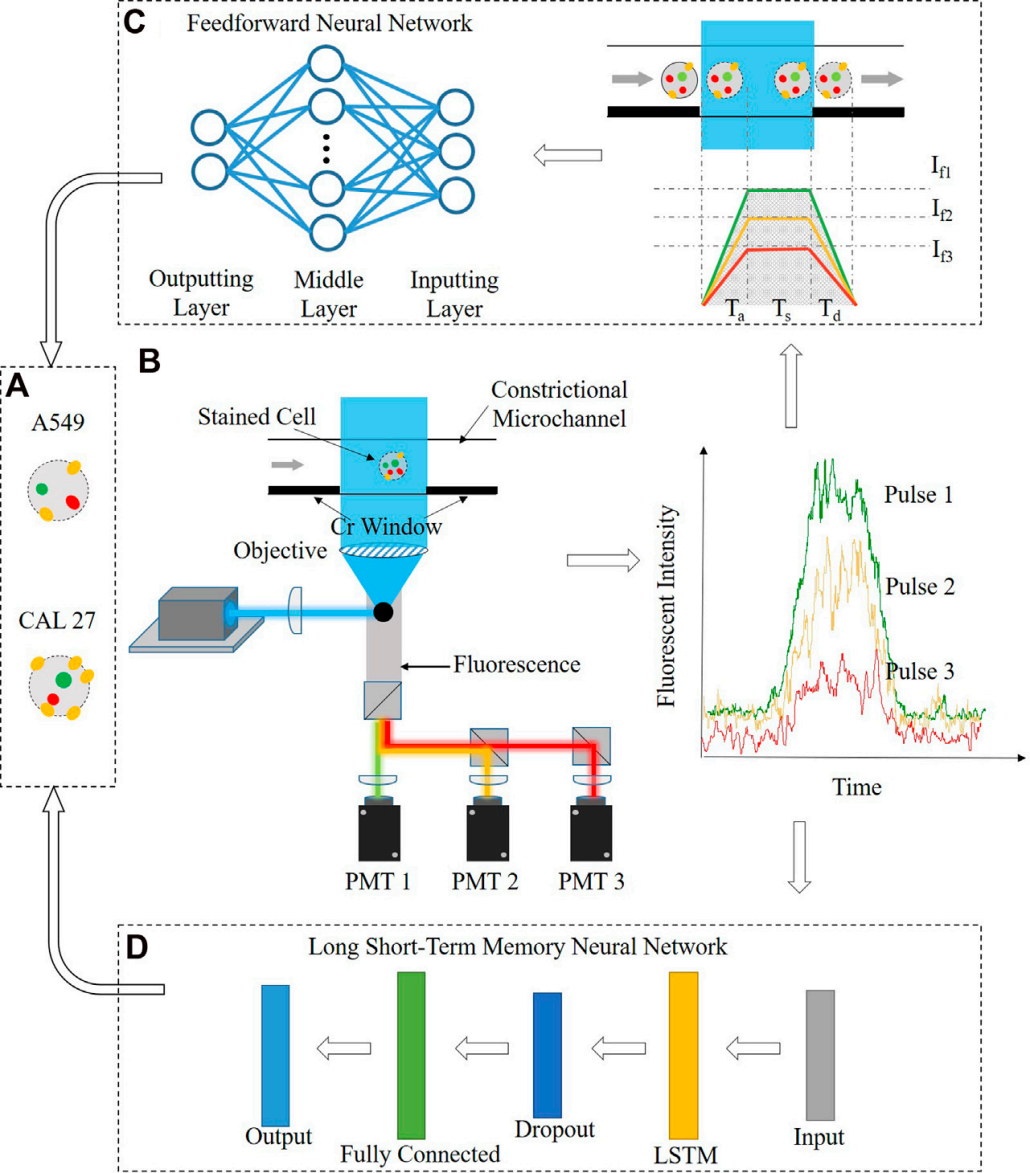
Working flow chart of the constrictional microchannel and the recurrent neural network in single-cell protein analysis, including **(A)** fluorescent acquisition based on fluorescent flow cytometry, leveraging constrictional microchannels, **(B)** cell-type classification via the feedforward neural network, where fluorescent pulses were translated into protein numbers based on the calibration model enabled by the constrictional microchannel, and **(C)** cell-type classification via the recurrent neural network, where fluorescent pulses were processed by the long short-term memory (LSTM) neural network.

Figure 1 shows the working flowchart for the cell-type classification based on fluorescent intensities of stained cells bound with fluorescence-labeled antibodies including fluorescent acquisition enabled by fluorescent flow cytometry, leveraging constrictional microchannels and cell-type classification via the feedforward or recurrent neural networks.

In fluorescent acquisition, individual cells were stained with three-type antibodies labeled with fluorescent probes (Figure 1A) and they were injected into the constrictional microchannel, which was excited by a laser beam and the fluorescent signals of the cells were detected using photomultiplier tubes (see Figure 1B; Supplementary Figure S1). In cell-type classification via the feedforward neural network, fluorescent pulses were first translated into single-cell protein numbers, leveraging calibration curves formed by flushing gradient fluorescence-labeled antibody solutions through constrictional microchannels with fluorescence samples (see Figure 1C). Then, quantified three-type single-cell protein levels of two cell types were applied to the two-layer feedforward neural network for leukocyte differentiation (see Figure 1D). In cell-type classification via the recurrent neural network, six fluorescent pulses of two cell types were directly applied to the six-layer LSTM neural network for differentiation (see Figure 1E).

### 2.2 Cell culture and treatment

The human non-small-cell lung cancer cell line (A549) was bought from Biology-Medicine Cell Resources of China, and the human tongue squamous cell carcinoma cell line (CAL 27) was a gift from the Peking University Hospital of Stomatology. Culturing reagents of Roswell Park Memorial Institute 1640 and Dulbecco’s modified eagle medium supplemented with sera and antibiotics were bought from Thermo Fisher, United States. Materials used for cell treatment including fixation (paraformaldehyde, PFA), membrane permeabilization (triton), and blocking (albumin) were purchased from the Sigma-Aldrich, United States. Fluorescence-labeled antibodies used for cell staining included β-actin-FITC and EpCAM-PE antibodies from Abcam, United Kingdom, and β-tubulin-PerCP antibody from Novus, United States. Positive photoresist from the AZ Electronic Materials, United States, negative photoresist of SU-8 from Microchem, United States, and elastomer of polydimethylsiloxane (PDMS) from Dow Corning, United States, were used for fabricating constrictional microchannels.

Membrane and intracellular staining of single cells was conducted by following well-established approaches including key steps of fixation, membrane blocking, membrane staining, membrane permeabilization, intracellular blocking, and intracellular staining. To be more specific, 1) in fixation, A549 and CAL 27 cell suspensions were incubated with 4% PFA solution for a quarter at 4°C; 2) in membrane blocking, 5% BSA was added for half an hour at 25°C; 3) in membrane staining, EpCAM antibodies with PE were diluted 100 times for bounding membrane proteins of cells for half an hour at 37°C; 4) in permeabilization, 0.05% triton X-100 was then added and incubated a quarter at 4°C; 5) in intracellular blocking, the same treatment was repeated as step 2; 6) in intracellular staining, β-actin antibodies with FITC and β-tubulin antibodies with PerCP were diluted 100 times for bounding intracellular proteins of cells for 4 h at 37°C. Following each procedure, a three-time rinse with 0.5% BSA was conducted to fully remove residual solutions.

### 2.3 Design and fabrication of constrictional microchannels

When the diameters of cells (∼15 μm) were taken into consideration, the geometrical dimensions of constrictional microchannels were determined as a height of 20 μm and a depth of 20 μm, which was modified from a previous study (Liu et al., 2020). The constrictional microchannel designed in this study was fabricated based on conventional fabrication techniques of microfluidics, which included hard lithography of SU-8, soft lithography of PDMS, chromium sputtering, and PDMS-glass bonding.

To be more specific, this constrictional microchannel was composed of a layer of patterned PDMS and a glass layer patterned with chromium windows. To fabricate the PDMS layer with patterned microchannels, the PDMS casting mold was prepared by the rotating bilayer SU-8 photoresist on top of a substrate, exposed with development. Briefly, to form the first layer of the constrictional microchannels with a height of 20 μm, the photoresist of SU-8 25 was spun on a bare substrate, soft baked, exposed with contact, and post-baked without development. Then, the SU-8 25 photoresist was applied, exposed with alignment, and developed to form microchannels (30 μm height) for cell traveling. For the step of soft lithography, the Sylgard 184 base and curing agents with the weight in the ratio of 15:1 were mixed, followed by vacuuming, pouring, and crosslinking.

As for the quartz layer with patterned chromium windows, after sputtering a layer of chromium on the quartz slide, the AZ1500 photoresist was spin coated, soft-baked, exposed, and developed. The patterned photoresist was transferred to the chromium layer based on chromium etching, followed by the removal of the residual photoresist and coating of a PDMS film with a height of 1 μm.

Lastly, after surface activation using the treatment of oxygen plasma, the microfabricated polymer layer composed of detection channels and the patterned quartz substrate composed of chromium windows were wetted with deionized water, aligned, and bonded together under a microscope. The assembled constrictional microchannels were kept on a hotplate to evaporate residual water and increase bonding strength.

### 2.4 Fluorescent acquisition

Key procedures of fluorescent acquisition were summarized as follows. Initially, a 150-mW laser was used for photobleaching with 20 min (OBIS, Coherent, United States) under the 10X objective lens of IX 83 from Olympus, Japan. After filling buffer solution of PBS with 0.5% BSA, single cell-bound antibodies with fluorescent probes (β-actin antibody of FITC, EpCAM antibody of PE, and β-tubulin antibody of PerCP) were injected into the constrictional microchannel. In order to drive single cells to travel continuously through the microchannel, PACE-5000 from GE Druck, United States, was used to generate a pressure of −1 kPa from the outlet.

A light source (488 ± 2 nm in wavelength and 5 mW in power) was used to excite fluorescent signals, when stained cells or gradient antibody solutions were passed through the constrictional microchannel. For fluorescent detection, PMTs of H10723-01 coupled with a bandpass filter of 534/30 nm, H10723-20 coupled with a bandpass filter of 575/25 nm, and H10722-20 with a bandpass filter of 692/40 nm from Hamamatsu, Japan, were used to detect three-channel fluorescence signals for FITC, PE, and PerCP probes, respectively. After signal captures by PMTs, a synchronous data acquisition card (USB-6349, National Instrument, United States) was used to sample signals at a sampling rate of 500 kHz.

For the raw data, a 50-point median filter was processed to remove background noises produced by PMTs. After the filtering, mean values with standard deviations of the preliminary fluorescent signals were calculated. In order to identify each event, a peaking value higher than the threshold (mean + 3 × standard deviation) was recognized as an effective pulse of a stained cell.

### 2.5 Protein measurement and the feedforward neural network

In calibrations, under the same experimental conditions of stained cells, gradient solutions (1:10, 1:50, 1:100, 1:500, 1:1,000, and 1:5,000) of β-actin-FITC, EpCAM-PE, and β-tubulin-PerCP antibody were injected into the constrictional microchannel to collect calibration curves with linear fitting. Due to volume equivalence formed by the constrictional microchannel, a certain number of fluorescent molecule-conjugated proteins were converted into corresponding fluorescent intensities, and thus, the fluorescent pulses were translated into a number of proteins. For detailed processes of protein quantification, please refer to the previous publication (Liu et al., 2020).

In this study, the feedforward neural network (MATLAB 2021b; MathWorks, United States) was leveraged to conduct cell-type classification based on aforementioned three-type protein expressions at the single-cell level. In this neural network, values of protein expressions were applied to the inputting layer, while the corresponding cell types (e.g., A549 vs. CAL 27) were used as the outputting layer. As to the middle layer, a neuron number of 50 was used for cell-type classification. In addition, the complete dataset (e.g., 10, 232 A549 and 16, 376 CAL 27) was divided into a training dataset (70%), a validation dataset (15%), and a testing dataset (15%) to produce a key parameter of “classification accuracy” in cell-type classification.

### 2.6 Recurrent neural network

As for comparison, fluorescent pulses of tumor cell lines of A549 and CAL 27 were processed by the recurrent neural network since connections among layered nodes in this network form a graph along the time sequence to exhibit temporal dynamic behaviors. Since the conventional recurrent neural network may result in vanishing gradients, when relatively long sequences were processed, LSTM was used in this study as a variation of the recurrent neural network, where a key unit of “forgetting gate” was included to process relatively long sequences with temporal gradients effectively kept.

Within six layers of LSTM, three parameters (e.g., batch size, learning rate, and neuron number) may deeply affect network performances. First, “batch size” represents the size of training data in iterations, where increases in batch size with corresponding decrease in iterations can produce decreases in performances of generalization and accuracies of classification but an increase in efficiencies of computation. Here, a group of batch sizes (e.g., 10, 100, and 1,000) were screened for optimization.

A high learning rate usually means a short computational time in convergence, while a small learning rate may require repeated computations without a convergence. In addition, as a hyperparameter, a high neuron number means a complex network with the concerns of overfitting, while a low neuron number may produce a oversimplified neural network. In this paper, varied values of learning rates (e.g., 0.01, 0.001, and 0.0001) and neuron numbers (e.g., 64, 128, and 256) were used for optimization.

## 3 Results and discussion

**FIGURE 2:**
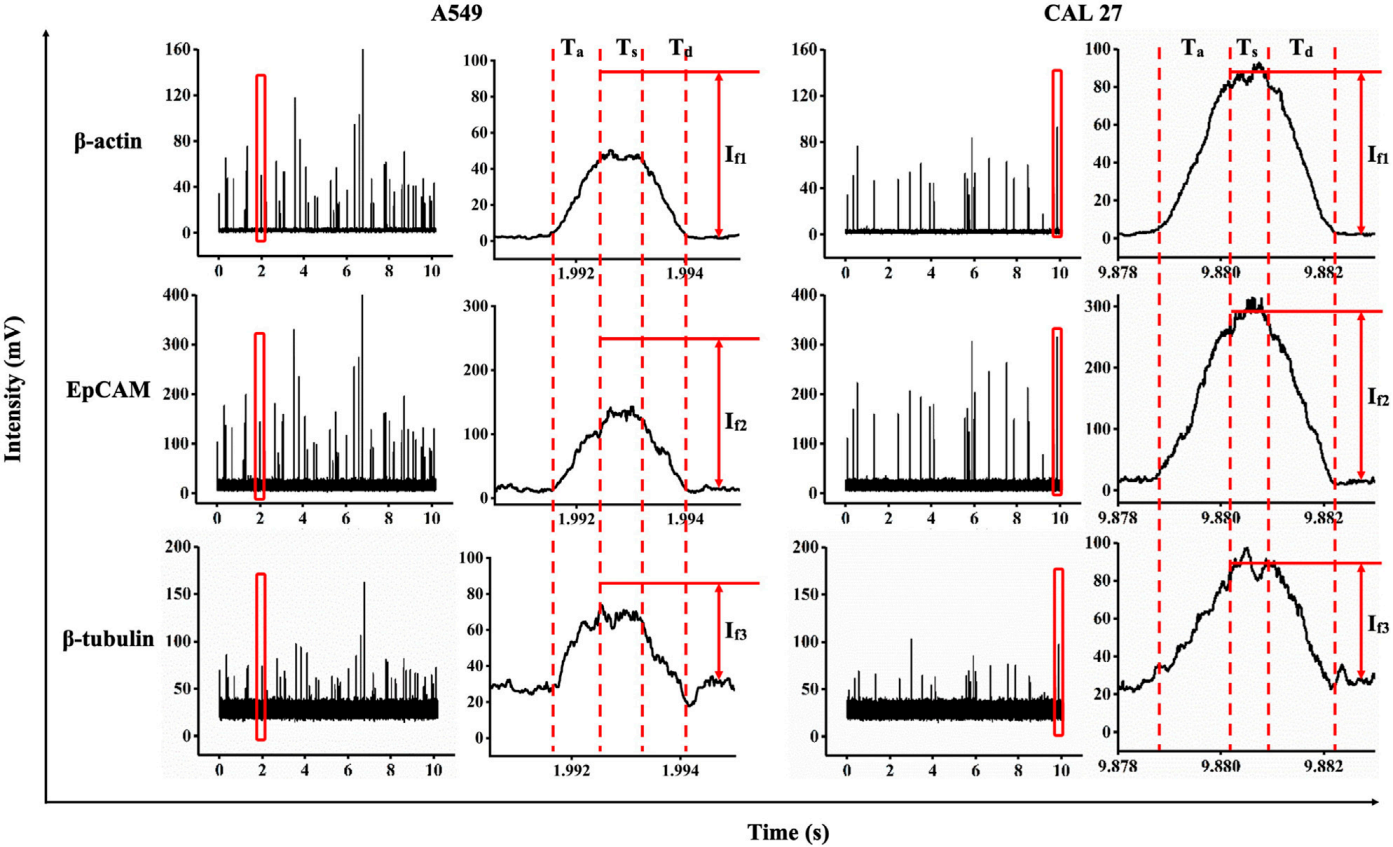
Fluorescent profiles (e.g., FITC-labeled β-actin antibodies, PE-labeled EpCAM antibodies, and PerCP-labeled β-tubulin antibodies) of individual A549 and CAL 27 cells traveling through constrictional microchannels.

As shown in Figure 2, data on individual A549 and CAL 27 cells were obtained with three time-coordinated pulses for the single cells, which represented the fluorescent levels of β-actin with a FITC probe, EpCAM with a PE probe, and β-tubulin with a PerCP probe. Similar to an arbitrary pulse, key indicators of T_a_, T_s_, and T_d_ and I_f1_, I_f2_, and I_f3_ were collected by fitting these pulses with ladder shapes.

The time width and traveling velocities of the cell passing through the constrictional microchannel were quantified as 3.00 ms ± 1.43 ms and 16.32 ± 13.12 μm/ms (A549, n_cell_ = 10,232) and 2.59 ms ± 0.84 ms and 17.16 ± 5.35 μm/ms (CAL 27, n_cell_ = 16,376). These parameters can, to an extent, reflect the throughput, which was mainly dominated by the magnitude of the driving pressure. Potentially, the driving pressure can be easily increased to further shorten the durations of traveling cells to less than 1 ms with throughputs significantly improved. For the raw fluorescent intensities of the pulses in the stable zones, I_f1_, I_f2_, and I_f3_ were calculated as 29.7 mV ± 17.4 mV, 80.5 mV ± 46.1 mV, and 25.4 mV ± 12.6 mV for A549 cells, and 44.0 mV ± 19.6 mV, 147.9 mV ± 66.0 mV, and 29.8 mV ± 14.0 mV for CAL 27 cells, respectively. These preliminary parameters, to an extent, reflect expressions of proteins during measurements.

**FIGURE 3:**
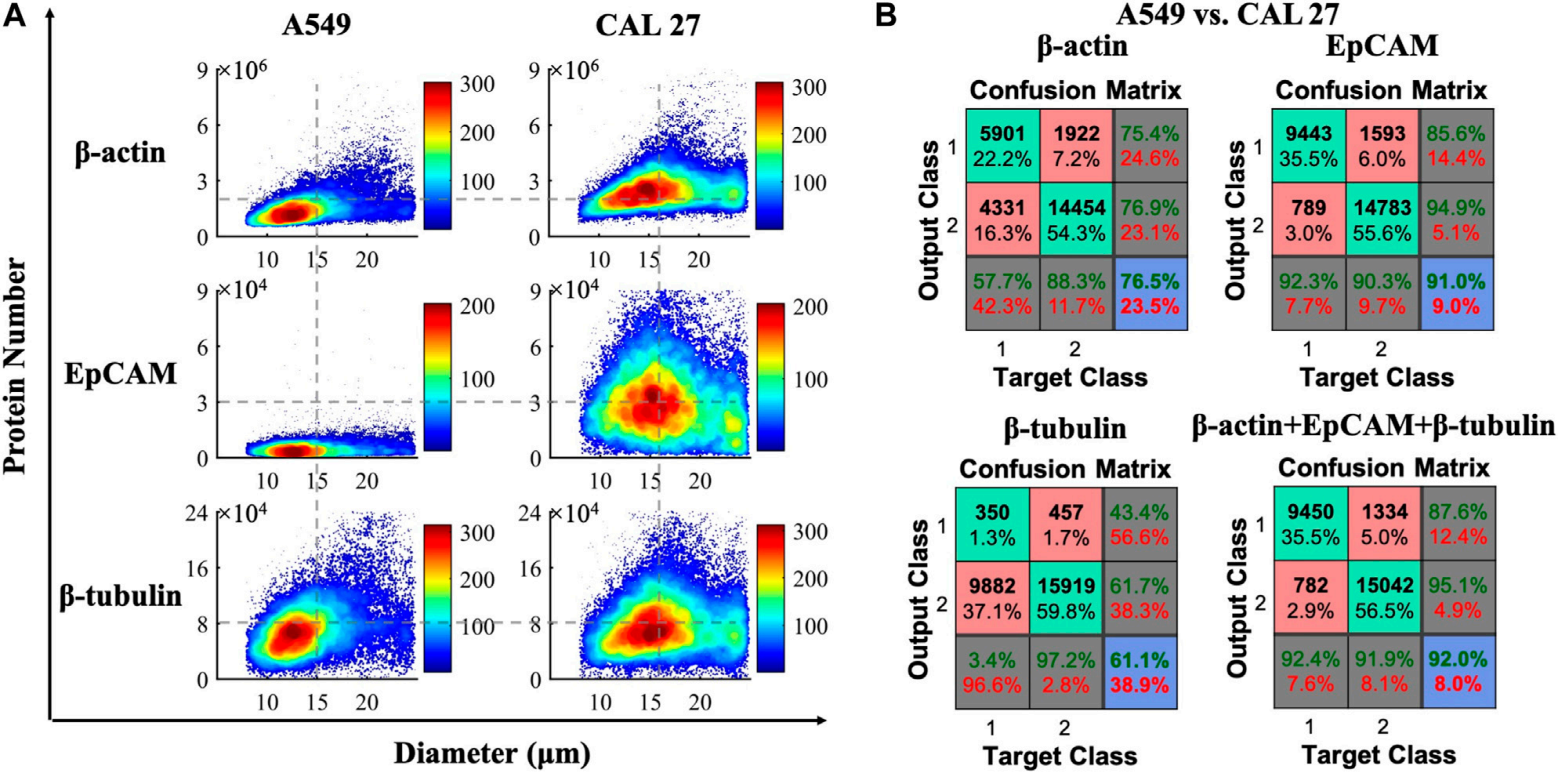
**(A)** Scatter plots of cell diameters and quantitative expressions of cytoplasmic proteins of β-actin/β-tubulin and membrane proteins of EpCAM from individual A549 (n_cell_ = 10, 232) and CAL 27 (n_cell_ = 16, 376). **(B)** Confusion matrix for classifying A549 and CAL 27 cells based on protein numbers and the feedforward neural network.

Similar to the scatter plots shown in Figure 3A, cell diameters and quantitative expressions of cytoplasmic proteins of β-actin/β-tubulin and membrane proteins of EpCAM from individual A549 or CAL 27 cells were obtained, leveraging calibration curves with compensations (see Table S1). To be more specific, the cell diameters were calculated as 15.2 μm ± 4.0 μm for A549 cells and 16.6 μm ± 4.0 μm for CAL 27 cells. For the validation of the results, A549 and CAL 27 cell suspensions observed by microscopic images were obtained as 15.9 μm ± 2.7 μm and 17.5 μm ± 2.0 μm, which was comparable with the results calculated by this method.

Protein expressions of β-actin, EpCAM, and β-tubulin at single-cell levels were quantified as 1.78 ± 1.06 × 10^6^, 0.56 ± 0.43 × 10^4^, and 8.11 ± 4.89 × 10^4^ for A549 cells (n_cell_ = 10,232), and 2.65 ± 1.19 × 10^6^, 3.47 ± 2.45 × 10^4^, and 8.61 ± 5.25 × 10^4^ for CAL 27 cells (n_cell_ = 16,376) (see Table S2). Compared with previous studies of protein numbers for β-actin (∼10^6^ per cell) and β-tubulin (∼10^4^ per cell) (Li et al., 2018b; Liu et al., 2020), the results of the numbers of β-actin and β-tubulin detected by this method were comparable. Furthermore, the number of EpCAM per A549 cells reported in this study fell into the same range with a previous value, which was ∼7,700 based on a microarray assay (Yang et al., 2016). Moreover, the expressions of EpCAM in A549 cells were significantly lower than those in CAL 27 cells, which was consistent with the previous studies (Wu et al., 2019; Wu et al., 2020).

Figure 3B shows the classification accuracies between A549 and CAL 27 cells, according to protein numbers based on the feedforward neural network. More specifically, the classification accuracies were quantified as 76.5% (β-actin), 91.0% (EpCAM), 61.1% (β-tubulin), and 92.0% (β-actin, EpCAM, and β-tubulin). Although the classification accuracies based on the membrane and intracellular proteins reported in this study were much higher than the classification accuracy of 73.3% based on only three intracellular proteins (Liu et al., 2020), there was still a big gap from 100% classification, which needs further efforts in neural pattern recognition.

**FIGURE 4:**
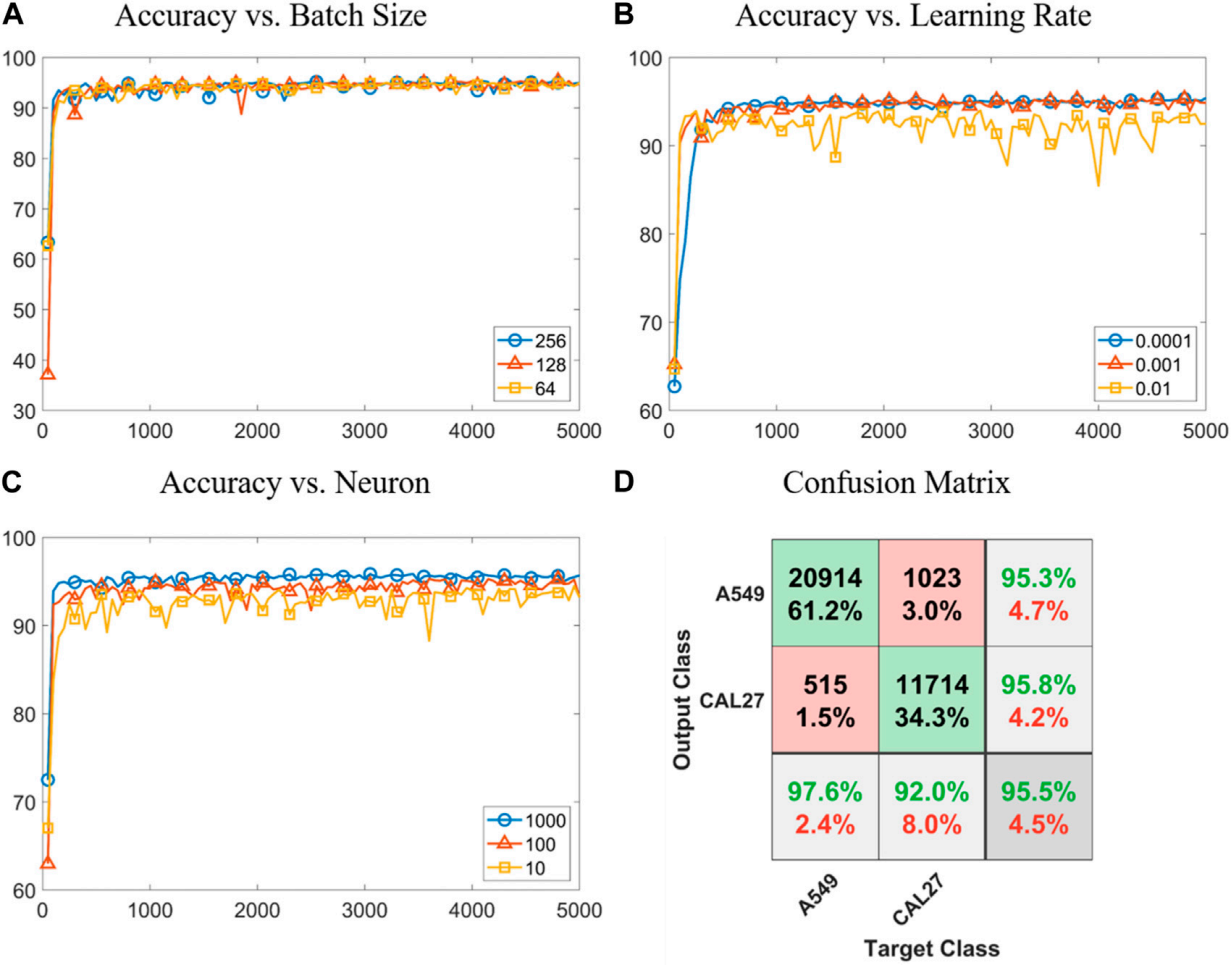
Results of classifying A549 and CAL 27 cells via the LSTM neural network. **(A–C)** Plots of classification accuracy vs. the iteration number with a varied “batch size,” “learning rate,” and “neuron number”. **(D)** Confusion matrix for classifying A549 and CAL 27 cells based on the optimized LSTM neural network.

Figure 4 shows the results of classifying A549 and CAL 27 cells via the LSTM neural network, specifically including plots of classification accuracy vs. the iteration number with a varied “batch size,” “learning rate,” and “neuron number” as well as a confusion matrix for classifying A549 and CAL 27 cells based on the optimized LSTM neural network. When the batch size was increased from 10 to 100 and then 1,000, steps of convergence and accuracies of classification were noticed to decrease. Due to the potential consideration of computing resources, here, 100 was chosen as the optimal batch size in the following utilization of LSTM (see Figure 4A).

In the optimization of the learning rate, when this parameter was first decreased from 0.01 to 0.001, the problem of repeating computations was relieved. As this parameter was further decreased from 0.01 to 0.0001, significant increases in durations of computation and convergence were located. Thus, a learning rate of 0.001 was further used in the following studies (see Figure 4B). If the neuron number was very high (e.g., 256), overfitting was observed, while if the neuron number was very low (e.g., 64), underfitting was found. When these issues were taken into consideration, a neuron number of 128 was used in this study (see Figure 4C).

When the optimized LSTM neural network was used to process fluorescent pulses, a classification accuracy of 95.5% (e.g., 95.6% of training with a sample size of 23,916, 95.4% of validation with a sample size of 5,125, 95.3% of testing with a sample size of 5,125, and 95.5% in total with a sample size of 34,166) was obtained in classifying A549 and CAL 27 cells (see Figure 4D). When the classification results of two tumor cell lines relying on the feedforward neural network vs. LSTM were compared, effective increases in classification accuracies from 92% to 96% were found. This was because in the feedforward neural network, only three-type protein expressions were used for cell-type classification, while in LSTM, fluorescent pulses were processed and more than 100 features were extracted for cell-type classification. Note that features extracted by LSTM had no physical meanings and they cannot be directly used for classifying other cell types without proper training.

## Data Availability

The original contributions presented in the study are included in the article/Supplementary Material; further inquiries can be directed to the corresponding authors.
