# Supplementary material for: Development of constrictional microchannels and the recurrent neural network in single-cell protein analysis
Source: Front Bioeng Biotechnol. 2023 May 3;11:1195940. doi: 10.3389/fbioe.2023.1195940 (PMC10190128; doi:10.3389/fbioe.2023.1195940)
Supplement: Supplementary file 1 [file DataSheet1.docx]

**Supplementary Materials**

Development of Constrictional Microchannel and Recurrent Neural Network in Single-Cell Protein Analysis

Ting Zhang^1,2*^, Xiao Chen^1,2*^, Deyong Chen^1,2,3^, Junbo Wang^1,2,3**^, and Jian Chen^1,2,3**^

^1^State Key Laboratory of Transducer Technology, Aerospace Information Research Institute, Chinese Academy of Sciences, Beijing, China

^2^School of Future Technology, University of Chinese Academy of Sciences, Beijing, China

^3^School of Electronic, Electrical and Communication Engineering, University of Chinese Academy of Sciences, Beijing, China

*Co-First Author

**Co-Corresponding Author

Junbo Wang (Aerospace Information Research Institute, Chinese Academy of Sciences), E-mail: jbwang@mail.ie.ac.cn

Jian Chen (Aerospace Information Research Institute, Chinese Academy of Sciences), E-mail: chenjian@mail.ie.ac.cn

**Fig. S1.** Schematics of a single cell passing through the detection structure with corresponding variations of measurement signals: (A) beginning, (B) entering, (C) passing and (D) leaving.

**Table. S1.** Calibration equations and compensation matrices of calibration curves.

| Calibration Equation | Channel 1 | Channel 2 | Channel 3 |
| --- | --- | --- | --- |
| β-actin | y=1.65×10^-5^x+2.52 | y=4.22×10^-5^x+17.25 | y=1.43×10^-6^x+31.45 |
| EpCAM | y=2.28×10^-6^x+2.22 | y=1.06×10^-3^x+12.53 | y=4.89×10^-5^x+30.18 |
| β-tubulin | y=2.32 | y=17.02 | y=2.33×10^-4^x+31.74 |
| Compensation Matrix | Channel 1 | Channel 2 | Channel 3 |
| β-actin | 100.00% | 256.29% | 8.67% |
| EpCAM | 0.21% | 100.00% | 4.62% |
| β-tubulin | 0 | 0 | 100.00% |

**Table. S2.** Summary of quantitative results of fluorescent intensities, cell diameters and protein numbers of single A549 and CAL 27 cells.

| Cell type | | A549 (N_cell_=10232) | | CAL 27 (N_cell_=16376) | |
| --- | --- | --- | --- | --- | --- |
| Parameter | | Mean | STD | Mean | STD |
| Ascending time (ms) | | 1.28 | 0.70 | 1.22 | 0.46 |
| Stable time (ms) | | 0.72 | 0.40 | 0.54 | 0.30 |
| Descending time (ms) | | 1.00 | 0.68 | 0.83 | 0.40 |
| Time width (ms) | | 3.00 | 1.43 | 2.59 | 0.84 |
| Travelling velocity (μm/ms) | | 16.32 | 13.12 | 17.16 | 5.35 |
| Preliminary fluorescent intensity (mV) | β-actin | 29.7 | 17.4 | 44.0 | 19.6 |
|  | EpCAM | 80.5 | 46.1 | 147.9 | 66.0 |
|  | β-tubulin | 25.4 | 12.6 | 29.8 | 14.0 |
| Compensated fluorescent intensity (mV) | β-actin | 29.7 | 17.4 | 44.0 | 19.6 |
|  | EpCAM | 4.5 | 4.6 | 35.2 | 25.9 |
|  | β-tubulin | 22.6 | 11.4 | 24.1 | 12.2 |
| Cell diameter (μm) | Detection | 15.2 | 4.0 | 16.6 | 4.0 |
|  | Image | 15.9 | 2.7 | 17.5 | 2.0 |
| Protein number | β-actin | 1.78×10^6^ | 1.06×10^6^ | 2.65×10^6^ | 1.19×10^6^ |
|  | EpCAM | 5.56×10^3^ | 4.32×10^3^ | 3.47×10^4^ | 2.45×10^4^ |
|  | β-tubulin | 8.11×10^4^ | 4.89×10^4^ | 8.61×10^4^ | 5.25×10^4^ |
